# Treatment of Bipolar Disorder by a Community Mental Health Service in a Rural Catchment Area in Greece: Treatment Engagement and Outcomes

**DOI:** 10.62641/aep.v53i5.1900

**Published:** 2025-10-05

**Authors:** Vaios Peritogiannis, Dimitra Moschou, Panagiota Gioti, Michailia Chlachla, Georgia Xiromerisiou

**Affiliations:** ^1^Mobile Mental Health Unit of the Prefectures of Ioannina and Thesprotia, Society for the Promotion of Mental Health in Epirus, 44445 Ioannina, Greece; ^2^Department of Psychiatry, University of Ioannina School of Medicine, 45110 Ioannina, Greece; ^3^Department of Neurology, Faculty of Medicine, School of Health Sciences, University of Thessaly, 41110 Larissa, Greece

**Keywords:** bipolar disorder, community mental health services, hospitalizations, rural areas, treatment engagement

## Abstract

**Background:**

The course of bipolar disorder (BD) may be disabling on several occasions, whereas management of BD may be challenging due to poor treatment adherence and high service-disengagement rates. Such challenges in the treatment of BD may be even more relevant in rural settings. In rural Greece, treatment of mental disorders may be almost exclusively delivered by the interdisciplinary Mobile Mental Health Units (MMHUs). The objective of the study was to explore treatment of BD by a MMHU in a rural setting in Greece.

**Methods:**

All medical records of BD patients that have been examined by the MMHU of Ioannina and Thesprotia (MMHU I-T) over a 17-year period (2007–2023) were assessed retrospectively. The studied outcomes were 1-year treatment engagement; treatment engagement at the study endpoint; and changes in hospitalizations and length of hospital stay in treatment-engaged patients.

**Results:**

From a total of 62 examined patients, data was analyzed for 48 cases. The 1-year engagement rate was 81.3%, which dropped to 52.1% at the study endpoint, with a mean follow-up of 7.2 ± 4.0 years. Treatment-engaged patients were more likely to receive a mood stabilizer than disengaged patients. In treatment-engaged patients a significant reduction in total and voluntary hospitalizations was observed, whereas involuntary admissions remained unchanged. Length of hospital stay was significantly reduced after treatment engagement.

**Conclusions:**

The results of the study are in line with previous research in Greek rural settings. The impact of the care by MMHUs on involuntary admissions in BD patients warrants further study. Future research should be multi-centered, with prospective design, and should address additional outcomes.

## Introduction 

Bipolar disorder (BD) is a severe and persistent, recurring mental illness associated with significant morbidity and disability [1]. Although the evolution and course of the disease may vary widely among patients, BD often negatively impacts the overall functioning of patients; causes troubles in the workplace; and difficulties in interpersonal relationships [2]. Furthermore, psychiatric comorbidities, such as anxiety disorders, substance abuse disorders, and personality disorders are common in BD and are associated with adverse clinical outcomes and poor functioning [3].

Along with the often-disabling course of BD, management may be further complicated by poor adherence to treatment and poor engagement in mental healthcare. Attendance of scheduled follow-up appointments is important for patients with severe mental illness, such as schizophrenia and BD, yet there is evidence that those patients are at a particularly elevated risk for poor adherence to recommended treatments and follow-up appointments [4]. In BD, clinical instability and the variety of symptoms render continuity of care after hospital discharge particularly important [5]. Poor treatment engagement may lead to exacerbation of symptoms and rehospitalization [6], whereas patients that regularly attend outpatient treatment have a lower risk of subsequent readmission [5]. Moreover, it was recently shown that higher continuity of care may significantly reduce the severity of symptoms and may significantly improve social functioning in patients with mental illness [7].

The challenges in the treatment of BD may be even more relevant in rural settings, since people living in rural communities experience significant barriers in accessing mental health care, including a shortage of psychiatrists and other behavioral health specialists [8]. Indeed, there is a paucity of research regarding the course and management of BD in such settings [9, 10]. In a previous study in the USA, that compared rural and urban patients with BD, it was found that rural patients were more likely to receive outpatient services for their mental health exclusively in the general medical sector; were more likely to use emergency room or hospital services than their urban counterparts, mainly for treatment of physical health problems; and were more likely to experience a manic episode and suicide attempts [11]. Another study in rural India reported that a considerable proportion (26%) of BD patients did not receive any treatment and that the pattern of course of the disorder was mania predominant [12]. A more recent study in rural Ethiopia also reported a high proportion of unipolar mania in BD patients and suggested that patients with unipolar mania had better social functioning and lower suicidality [13]. Other research in a low-resourced setting found that health‑related quality of life among patients with BD in rural Uganda was poor [14]. Finally, a recent study in the USA used an economic analysis to address the impact of tele-psychiatry in the management of severe and persistent mental illness, including BD in rural patients. The authors suggested that such programs may be cost effective even assuming small improvements in hospitalizations [15].

In rural Greece and in several of the numerous Greek islands, care for mental illness is almost exclusively delivered by locally based generic community mental health teams, the so-called mobile mental health units (MMHUs) [16]. The MMHUs are interdisciplinary teams that treat all mental disorders, but prioritize patients with severe and persistent mental illness, such as schizophrenia-spectrum disorders and BD [17]. It has been previously reported that MMHUs are effective in engaging patients with psychotic disorders to treatment [18] and in reducing hospitalizations and length of hospital stay in those patients [19]. Less is known regarding treatment of patients with BD by the MMHUs in rural settings in Greece. Indeed, there is only a previous study in insular Greece, which comprised a mixed population of patients with psychotic disorders and BD, which recorded significant reduction in hospitalizations and length of hospital stay across all diagnoses [20]. The objective of the present study was therefore to explore the care of patients with BD by a MMHU in rural Greece, focusing on treatment engagement and psychiatric hospitalizations.

## Materials and Methods 

### The Study Setting

The MMHU of the prefectures of Ioannina and Thesprotia (MMHU I-T) delivers services in rural and remote, mountainous areas in the Epirus region, Northwest Greece, for a population grossly estimated at 100,000 inhabitants. The interdisciplinary, generic community mental health team delivers evidence-based care with the use of a case management approach. Treatment includes pharmacotherapy; psychosocial interventions, such as psychoeducation and support for patients and their caregivers; enhancement of selfcare and independent living; and social work. Such multifaceted care is particularly relevant in the treatment of severe and persistent mental illness, such as schizophrenia spectrum disorders and BD [21].

### Study Design

This is a retrospective, 17-year observational study (March 2007–December 2023) comprising patients with a diagnosis of BD (F31), according to the International Classification of Mental and Behavioral Disorders-10th revision (ICD-10) [22], that attended the MMHU I-T. Data was retrieved from patients’ charts and involved demographic (gender, age, etc.) and clinical information (illness duration, history of alcohol/substance abuse, etc.). Patients were included in the study based on a diagnosis of BD, according to ICD-10; and age ≥18 years at first examination by the MMHU I-T. Exclusion criteria included: patients who were referred to the MMHU I-T for prescription refill or certificate administration; co-morbid organic brain disorder; and severe mental retardation. Patients who died over the follow-up period and those that moved out of the catchment area were also excluded from analysis.

### Service Engagement Assessment 

To assess engagement of BD patients with the MMHU I-T all cases that initially attended the service were considered. Service engagement was defined as regular attendance of scheduled follow-up appointments. Follow-up of patients with severe mental illness, such as BD, is usually based on monthly or bimonthly appointments, but frequency may vary according to patients’ needs and mental state. When patients missed consecutive appointments, or failed to attend at least 80% of scheduled appointments, they were rated as disengaged. To assess engagement over time, two distinct periods were defined: 1-year treatment engagement and engagement at study endpoint. One-year engagement was defined when patients completed 1-year follow-up at any point of the 17-year study interval; whereas engagement at study endpoint was defined when patients were engaged to treatment with the MMHU I-T in December 2023.

### Assessment of Differences in Hospitalizations and Length of Hospital Stay in Treatment-engaged Patients

Data on hospitalizations and length of hospital stay was retrieved from the charts of patients that were still engaged in treatment with the MMHU I-T in December 2023 and was further analyzed. A mirror image, pre-post comparison design, which has previously been used in research [23], was applied for the estimation of potential differences in psychiatric hospitalizations prior and after treatment engagement of patients with the MMHU I-T. Accordingly, for each patient, comparison was made for the same interval prior and after engagement to treatment with the MMHU I-T. The study was conducted in accordance with the Declaration of Helsinki. Ethical approval for the study conduct was granted by the Institutional Review Board of the Society for the Promotion of Mental Health in Epirus (Δ.2./ 25-7-2024). Patient consent was waived due to the non-interventional nature of this study.

### Statistical Analysis

Categorical variables are presented as frequencies and percentages, whereas continuous variables are summarized as mean ± SD. The dependent variables (outcomes) of the present study were hospital admissions, length of hospital stay, voluntary hospital admissions and involuntary hospital admissions. Due to the small study sample, we employed non-parametric statistical tests that make no assumption that our data is generated from a particular distribution. To determine the effect of treatment-engagement with the MMHU I-T Wilcoxon signed ranked tests were applied comparing outcomes before and after the intervention. Additionally, contingency tables (cross-tabulations) using the chi-square test and the Mann-Whitney non-parametric test for independent samples were used to evaluate the distribution of categorical and continuous independent variables respectively across engaged and disengaged patients. In all analyses *p* < 0.05 was declared as statistically significant. All analyses were carried out using SPSS statistical software version 25 (IBM Corp., Armonk, NY, USA).

## Results

**Fig. 1.**
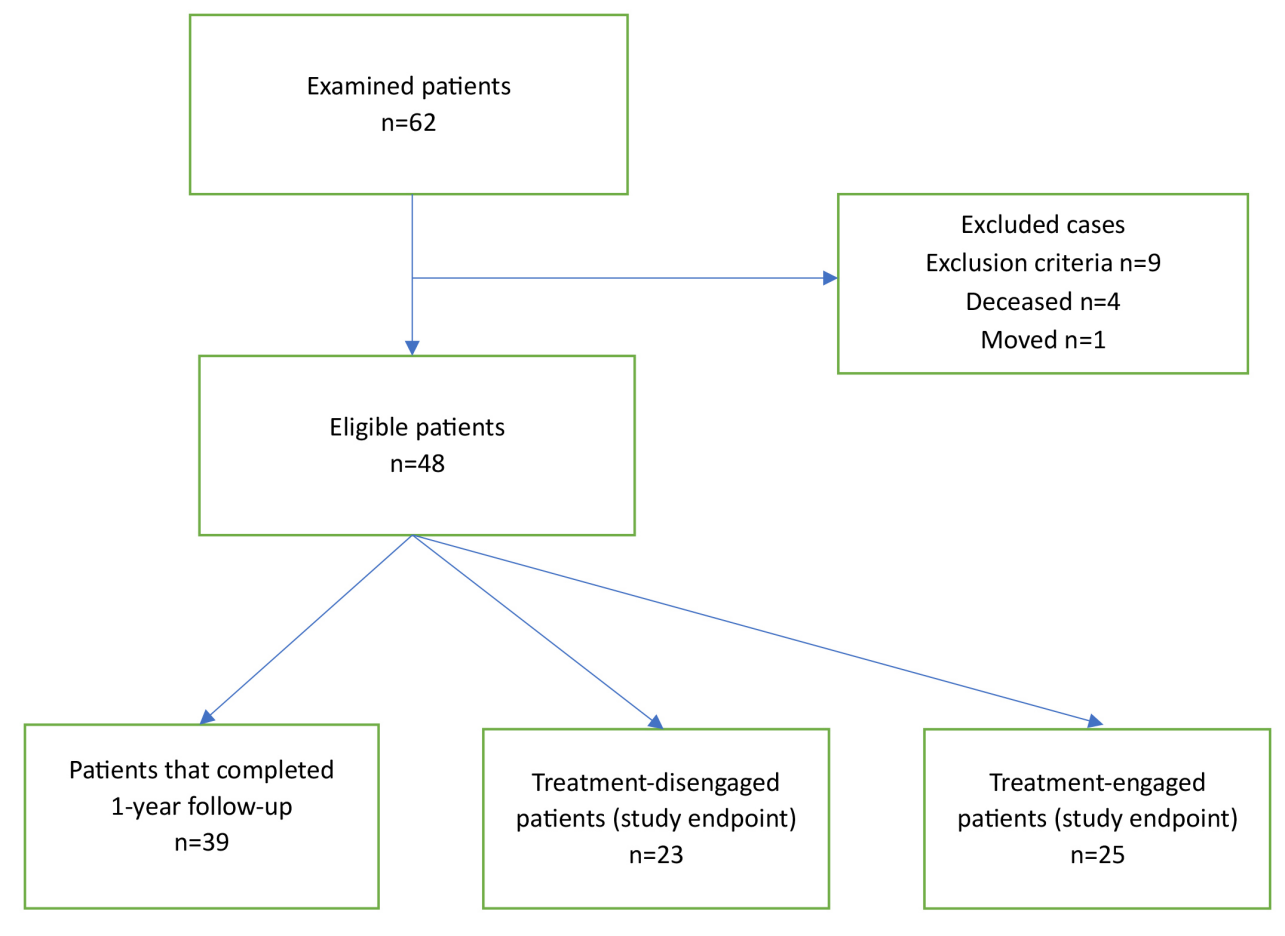
**Flowchart of bipolar patients’ selection**.

From a total of 62 patients with BD, that had been examined by the MMHU I-T over the 17-year study period, 48 eligible cases were considered for analysis. Fig. 1 depicts the flowchart of patients’ selection.

### Engagement to Treatment and its Correlations

The 1-year engagement rate was 81.3%, whereas at study endpoint engagement rate dropped to 52.1%, with a mean follow-up of 7.2 ± 4.0 years. The demographic and clinical characteristics of the patients’ sample are presented in Table 1. It appears that the two patient groups did not differ in most of the examined clinical and demographic variables. There was a statistically significant difference in mood stabilizer prescription, with engaged patients having been prescribed such treatment more often.

**Table 1.** **Demographic and clinical characteristics of engaged and disengaged patients**.

		Engaged (n = 25)	Disengaged (n = 23)	Statistical test	*p*-value
Age at first examination (years)		52.3 ± 10.9	56.5 ± 12.9	Z = –1.023	0.306
Gender					
	Male	9 (36.0%)	8 (34.8%)	χ^2^ = 0.008	0.930
	Female	16 (64.0%)	15 (65.2%)		
Illness duration at first examination (years)		25.8 ± 14.0	22.6 ± 14.5	Z = –1.105	0.269
Caregiver					
	No	9 (36.0%)	3 (13.0%)	χ^2^ = 3.367	0.067
	Yes	16 (64.0%)	20 (87.0%)		
Disability pension					
	No	7 (28.0%)	12 (52.2%)	χ^2^ = 2.927	0.087
	Yes	18 (72.0%)	11 (47.8%)		
Physical morbidity					
	No	7 (28.0%)	7 (30.4%)	χ^2^ = 0.034	0.853
	Yes	18 (72.0%)	16 (69.6%)		
Substance/alcohol abuse					
	No	15 (60.0%)	15 (65.2%)	χ^2^ = 0.139	0.709
	Yes	10 (40.0%)	8 (34.8%)		
Treatment regimen (last recorded)					
	Antipsychotic	21 (84.0%)	16 (69.6%)	χ^2^ = 1.143	0.235
	Antidepressant	8 (32.0%)	5 (21.7%)	χ^2^ = 0.639	0.424
	Mood stabilizer	18 (72.0%)	10 (43.5%)	χ^2^ = 4.009	0.045
	Benzodiazepine	13 (52.0%)	11 (47.8%)	χ^2^ = 0.083	0.773

### Changes in Hospitalizations and Length of Hospital Stay in Treatment-engaged Patients

**Fig. 2.**
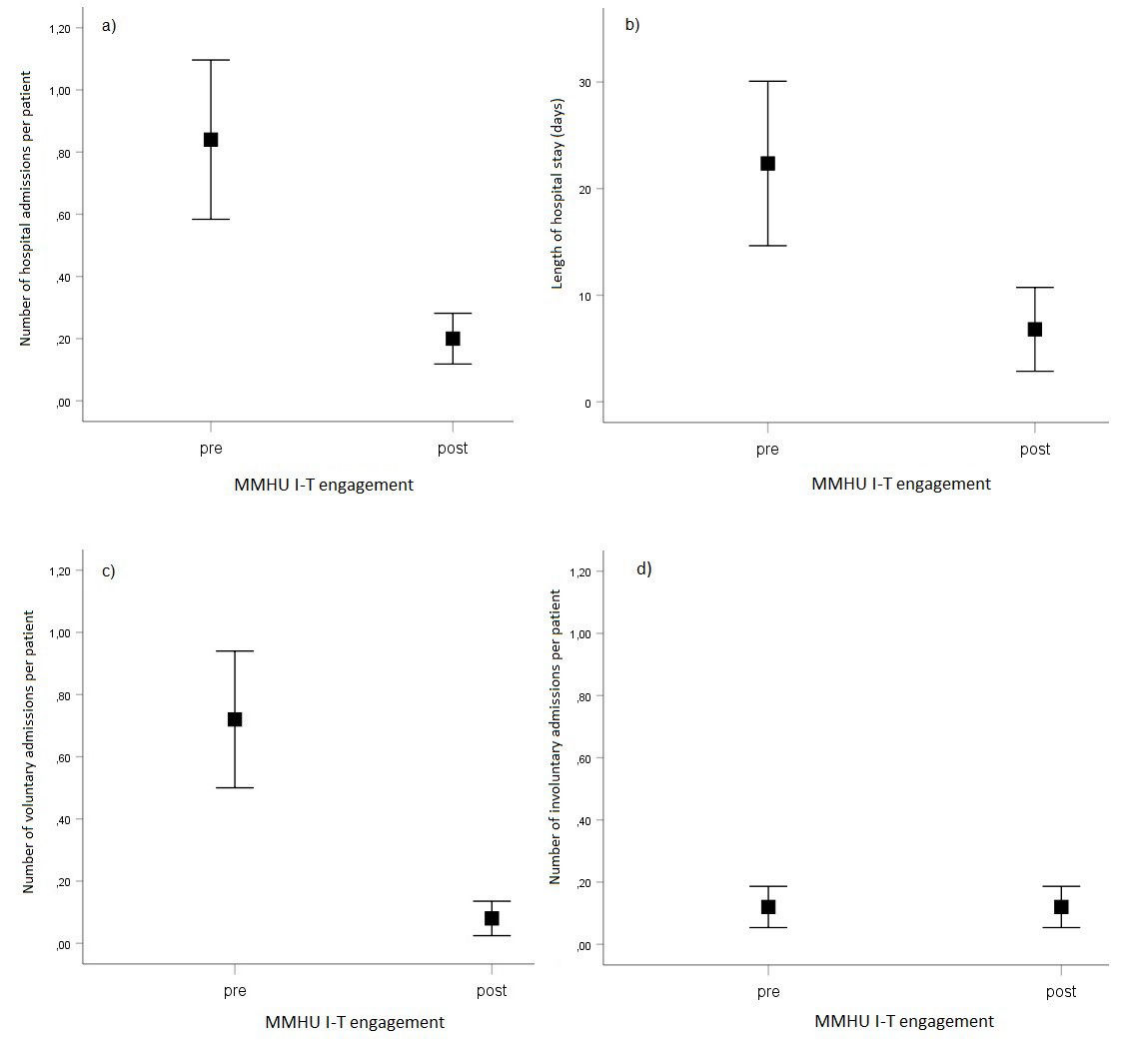
**Changes in hospitalizations and length of hospital stay in treatment-engaged bipolar patients**. (a) total hospitalizations; (b) length of hospital stay; (c) voluntary admissions; (d) involuntary admissions. MMHU I-T, The MMHU of the prefectures of Ioannina and Thesprotia.

Patients that were rated as treatment-engaged at the study endpoint (n = 25) were further analyzed. The mean age of those patients at the study endpoint was 61.1 ± 12.0 years, with a mean illness duration of 32.4 ± 14.8 years. Our analyses on the outcomes revealed reduced mean hospital admissions post-engagement compared to pre-engagement (Z = 2.000, *p* = 0.014). In addition, the mean length of hospital stay was significantly less post-engagement compared to pre-engagement (Z = 2.497, *p* = 0.013). The number of voluntary hospital admissions post-engagement was significantly reduced compared to pre-engagement (Z = 2.714, *p* = 0.007). Involuntary admissions did not differ significantly between pre- and post-engagement periods (Z = 0.012, *p* = 0.981). Differences in mean hospital admissions, mean length of hospital stay and mean voluntary and involuntary hospital admissions after treatment-engagement with the MMHU I-T are shown in Fig. 2.

## Discussion

The present study adds to the limited research on the treatment of BD in rural settings; and expands the findings of a previous study in insular Greece, that comprised a mixed population of patients with psychotic disorders and BD and recorded significant reduction in hospitalizations and length of hospital stay in patients regularly attending a MMHU [20].

Over the 17-year study period only 62 cases of BD were examined by the MMHU I-T. This number is rather small and should be commented on. In the study by the MMHU of Zakynthos, Kefalonia and Ithaca that assessed treatment outcomes in patients with severe and persistent mental illness, the number of BD cases had been almost 4-fold lower than the respective number of patients with psychotic disorders [20]. Moreover, a recent study by the MMHU I-T showed that first-contact patients with mood disorders, such as BD, were less likely to engage in treatment, compared to patients with schizophrenia-spectrum disorders [24]. These observations suggest that patients with BD may have more difficulties engaging in treatment with a MMHU in the rural setting than their counterparts suffering a psychotic disorder. The reason is not clear, but the fluctuating course of BD could account for the relatively low attendance rates. Or the more benign outcome of BD compared to schizophrenia-spectrum disorders [25, 26, 27] could explain the higher disengagement rates, as several patients may not need the comprehensive treatment delivered by the MMHUs. Presumably, adequate- and high-functioning patients could feel stigmatized in the rural setting [28] and avoided attending a local community mental health service. Such patients and their families would prefer to be examined by private practice clinicians or in the outpatient clinics of urban public hospitals.

A finding that warrants further consideration is the decline of treatment engagement over time. It appears that the 1-year attendance rate of BD patients is rather high (81.3%), although the long-term relevance of this finding is unclear, given the chronic course of BD. However, treatment engagement declined substantially to 52.1% at study endpoint, with a mean follow-up of 7.2 ± 4.0 years. This finding corresponds to previous research in community treatment settings, which suggested that continuity of care in severe mental illness may decline over time [29] and may have important implications for clinical practice, because continuity of care has been associated with favorable outcome in patients with mental illness [7]. It is interesting to note that previous research has shown that almost 30% of patients with BD failed to attend a 30-day mental health follow-up outpatient visit post-discharge [5], highlighting the difficulties of this patient-population to engage in treatment.

No differences between treatment engaged and disengaged patients with BD in the examined clinical and demographic variables were recorded, except for the prescription of mood stabilizers, that was more common in engaged patients. Drug treatment of BD may often be complex and may require combination of different classes of drugs, including mood stabilizers [30]. It seems that disengaged patients received sub-optimal drug treatment, however the reason is unknown. It could be argued that several patients may have a negative attitude toward drug treatment and toward complex treatment regimens, which may imply a general tendency to treatment non-adherence. There is recent evidence that a substantial proportion of patients with BD do not receive their medications as prescribed [31]. Subsequently, such patients may be less willing to attend follow-up appointments in the long-term and finally disengage from treatment. Indeed, it has been shown that irregular follow-up was associated with psychotropic medication non-adherence [32]. However, due to the small sample size, conclusions cannot be drawn, and further study is warranted to address any differences in treatment regimen between engaged and disengaged BD patients.

In patients who were engaged in treatment at the endpoint of the study, a significant reduction in total hospital admissions was observed, mostly accounted for by the significant reduction in voluntary admissions, whereas involuntary admissions did not differ between pre- and post-engagement periods. Length of hospital stay was significantly reduced after engagement in treatment with the MMHU I-T. These findings correspond to previous research in Greek rural settings that mostly involved patients with schizophrenia-spectrum disorders, but also patients with BD [19, 20]. However, the present findings differ from previous research regarding involuntary admissions, as no differences were detected after treatment engagement. Treatment of BD patients in rural settings may be challenging, especially for those with a history of involuntary admissions, probably due to manic episodes. It has been suggested that treatment acceptance and adherence are often affected by diminished insight during manic episodes; accordingly, inpatient treatment may be most appropriate in mania, and involuntary admission may be necessary when treatment cannot be provided otherwise [33].

## Limitations and Strengths

The present study has some limitations that should be considered. Firstly, it involves a single MMHU and a single catchment area, whereas the sample of patients is rather small, which causes margins of significant difference for several parameters. Moreover, due to the retrospective design of the study, some information may have been missing. Subsequently, generalizability of findings may be uncertain. Also, the study examined treatment of BD patients in a rural setting, and it lacks a control setting, for example urban regions. However, the present study also has some strengths that render the results relevant. It is an original study in a rural area that adds to a limited literature. Furthermore, it replicates the results of previous research [20], thus strengthening the evidence on the effectiveness of the MMHU model in the treatment of severe mental illness in rural areas [34].

## Implications for Clinical Practice

The present study may have potential implications for clinical practice and mental health policy in rural areas. It appears that treatment of BD patients by MMHUs in rural Greece may reduce inpatient care use, yet a substantial proportion of patients disengage from long-term treatment. Accordingly, efforts should be made toward improving treatment engagement. Previous research in rural Greece has suggested that an Assertive Community Treatment (ACT)-like approach for patients with severe mental illness that are difficult to engage to treatment could address this issue and improve symptomatology and functioning [23]. Subsequently, certain patients with bipolar disorder should be better assigned to ACT-like care, where available, to improve adherence and outcome.

## Conclusions 

The present study adds to a limited literature regarding treatment of severe mental illness in rural settings and replicates the results of previous research; however, further research on the impact of the care delivered by generic MMHUs in rural settings on involuntary admissions of patients with BD is warranted. Future research should have a prospective design, with the participation of as many MMHUs as possible, that would ensure the recruitment of large samples of patients in order to properly address the effectiveness of such care in the treatment of BD. Other outcome aspects, such as symptomatology and functioning should also be studied.

## Availability of Data and Materials

Not applicable.
